# Three‐point Method to Guide the Tibial Resection and Component Placing in Total Knee Arthroplasty

**DOI:** 10.1111/os.12693

**Published:** 2020-06-22

**Authors:** Ming‐yang Liu, Hai‐bo Wang, Shi‐wei Liu, Guan‐peng Zhang, Jian‐guo Liu, Chen Yang

**Affiliations:** ^1^ Department of Orthopaedic Surgery The First Hospital of Jilin University Changchun China

**Keywords:** Component, Orientation, Tibial resection, Total knee arthroplasty

## Abstract

**Objective:**

To introduce a three‐point method combining the midpoint of the posterior cruciate ligament (PCL), the midsulcus of the tibial spines, and the midpoint of the anterior cruciate ligament (ACL) to determine appropriate tibial resection and component placing during TKA and to compare this method with Insall's 1/3 method.

**Methods:**

A consecutive series of 128 knees that underwent TKA from January 2015 to August 2018 were analyzed. In one group (64 knees), the medial 1/3 of tibial tubercle (the Insall's traditional method) was used for tibial component alignment. In the other group (64 knees), the three‐point line connecting the midpoint of the PCL, the midpoint of the tibial spines, and the midpoint of the ACL was used for tibial component alignment. Both groups used the anterior tibial tendon as the distal reference for tibial resection. The coronal alignment error of the tibial component was determined by the angle between the line parallel to the tibial component platform and the tibial mechanical axis measured on postoperative radiograph. The axial rotation error of the femoral or tibial component was the intersection angle between the transepicondylar axis (TEA) and a line tangent to the posterior edge of the femoral or tibial component measured on CT. The coronal and axial alignment errors were compared between the two groups.

**Results:**

The average coronal alignment error of the tibial component in the three‐point method group was 0.2° ± 1.4° versus − 0.9° ± 1.8°in the Insall's 1/3 method group (*P* < 0.001), and the mean absolute value in the three‐point method group reduced by 37.3% compared to Insall's traditional method group. The average axial rotation error of the femoral component was 0.2° ± 1.2° in the three‐point method group versus − 1.1° ± 1.7° in the Insall's 1/3 method group (*P* < 0.001), and the mean absolute value in three‐point method group decreased by 43.9% compared to Insall's traditional method group. The average axial rotation error of the tibial component was 0.4° ± 1.4° versus − 1.4° ± 1.8° in the Insall's 1/3 method group (*P* < 0.001), and the mean absolute value in the three‐point method group reduced by 35.5% compared to the Insall's traditional method group. The rates of rotation outliers were significantly lower in the three‐point method group (*P* < 0.05).

**Conclusion:**

The line connecting the midpoint of the PCL, the midsulcus of the tibial spines, and the midpoint of the ACL could be used as the reference for the tibial resection and component placing. This method appears to be more accurate than Insall's 1/3 method. The results of this study provide a candidate method for component orientation with little error.

## Introduction

It is widely considered that appropriate coronal alignment and axial rotation of the tibial and femoral components is an important objective of total knee arthroplasty (TKA)^1^, ^2^, ^3^. Malalignment of the femoral and tibial components can result in polyethylene wear, abnormal patellar tracking, anterior knee pain, and aseptic loosening^4^, ^5^, ^6^. Especially on the tibial side, a non‐neutral resection will lead to misjudging the gap balancing, excessive soft‐tissue release, and rotation error for the femoral component^7^, ^8^, ^9^.

The transepicondylar axis (TEA) is generally used to determine the external rotation of the femoral component^10^. The corresponding tibial component is supposed to be perpendicular to the TEA to minimize impingement between the polyethylene post and the metal box^5^. However, the most reliable anatomical landmarks for component orientation on the tibial side remain controversial^3^, ^11^, ^12^. Several anatomical landmarks have been proposed for guiding the direction of the tibial component, including the medial 1/3 of the tibial tubercle, Akagi's line, the anterior and posterior cruciate ligament (APCL) line, and the midsulcus line^1^, ^2^, ^3^, ^13^. The traditional reference used in the proximal tibia is the medial 1/3 of the tibial tubercle that was proposed by Insall^13^. However, it seems to be “experiential” and lacking supportive evidence. Several studies show that Insall's line would lead to excessive external rotation of the tibial component^1^, ^3^, ^14^. Akagi's line was described as perpendicular to the TEA, but the authors did not explain why the TEA and the line connecting the midpoint of the posterior cruciate ligament (PCL) to the medial border of the tibial tubercle were orthogonal^1^. The APCL line was defined as a line connecting the stumps of the midpoint of the anterior cruciate ligament (ACL) and the PCL^3^. This direction ensures that the ACL and the PCL have no impingement on the intercondylar notch during knee flexion. It provides a reasonable explanation on APCL formation relative to the TEA, but its application might be limited because it is based on the residual stump of soft tissue^3^. Dalury^2^ describes a midsulcus line which connects the midsulcus of the tibial spines and the medial border of the tibial tubercle. However, its use would probably be limited in severe osteoarthritis patients whose tibial spines are worn away.

All of the above methods would be influenced by the osteophytes, worn tibial spines, obscure ligaments stumps, and tibial tubercle variation. A recently published systemic review recommended that a combination of more anatomical landmarks be used to ensure adequate tibial component rotation^12^. Interestingly, in a previous three‐dimension (3D) CT study, Yang *et al*.^3^ discovered that the midpoint of the PCL, the midsulcus of the tibial spines, and the midpoint of the ACL almost lay on the same line. This line was theoretically perpendicular to TEA^3^. Moreover, in another 3D CT study, Dalury and Aram^15^ found the line drawn along the midsulcus of the tibial spines and continued in the sagittal plane until the anterior tibia could achieve a resection surface within 3° of neutral mechanical alignment. Therefore, we hypothesized that correct tibial resection and component rotation could be achieved by using a three‐point line connecting the midpoint of the PCL, the midsulcus of the tibial spines, and the midpoint of the ACL.

The purpose of this study was: (i) to present the three‐point method for the determination of tibial resection and component placing during TKA; (ii) to evaluate the accuracy of coronal alignment of the tibial component and rotational alignment of femoral and tibial components using the three‐point method on postoperative radiographs and CT; and (iii) to compare the component orientation in patients using the three‐point method with patients using Insall's 1/3 method.

## Methods

### *Study Population*

Inclusion criteria: (i) osteoarthritis (OA) patients (64 knees) with varus knee who were undergoing TKA from June 2016 to August 2018; (ii) three‐point method combining the midpoint of the PCL, the midsulcus of tibial spines, and the midpoint of the ACL to determine tibial resection and component placing during TKA; (iii) patients (64 knees) using Insall's traditional 1/3 method during TKA from January 2015 to April 2016; (iv) coronal alignment error of the tibial component measured on postoperative long‐leg radiographs, axial rotation error of femoral component measured on postoperative CT, axial rotation error of tibial component measured on postoperative CT; and (v) retrospective study.

Exclusion criteria for this study were: (i) patients with congenital deformity of lower extremities; (ii) patients with genuflects valgus; and (iii) patients with previous knee joint surgeries. This study was approved by the institutional review board of our institute, and informed consent was obtained from all patients who participated in this study. The demographic and preoperative characteristics of patients are showed in Table 1.

**TABLE 1:** The demographic and preoperative characteristics of patients in the two groups (mean ± SD)

Characteristics	Three‐point method group	Insall's method group	*P*
Age (years)	68.1 ± 6.8	67.6 ± 6.2	NS
Gender (F/M)	36/19	37/18	NS
Side (L/R)	34/30	33/31	NS
BMI (Kg/m^2^)	25.2 ± 1.7	25.0 ± 1.6	NS
Height (cm)	162.2 ± 7.4	162.4 ± 7.0	NS
K‐L Grade (III/IV)	20/44	22/42	NS
Severity of varus (degree)	11.8 ± 3.8	12.0 ± 3.7	NS

BMI, body mass index; F, female; K‐L Grade, Kellgren–Lawrence grade; L, left; M, male; NS, not significant; R, right.

### *Surgical Technique*

#### *Anesthesia and Position*

All operations were performed under general anesthesia with the patients in supine position.

#### *Approach and Exposure*

All patients underwent midline incisions and medial parapatellar approaches. The surgeon made an incision down the center of the knee approximately 12 to 15 cm long, and then cut through deeper tissue, including the quadriceps tendon, and flipped over the patella to access the femur and tibia.

#### *Tibial Resection*

**Fig. 1:**
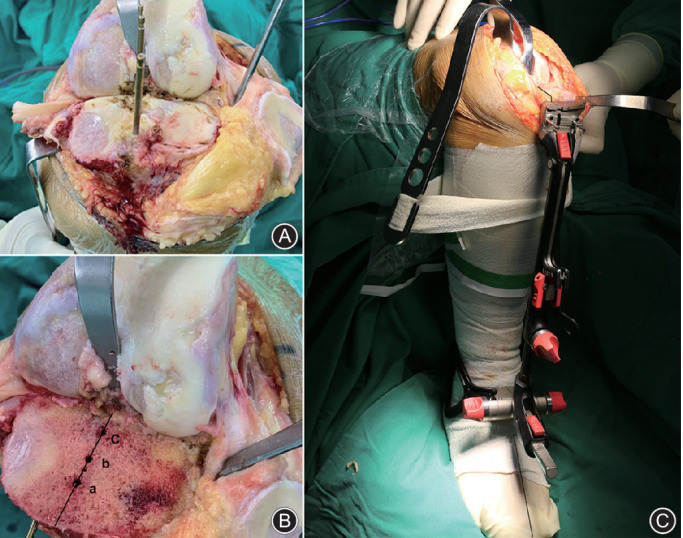
(A) Three nails were inserted into the anatomical landmarks. (B) a, midpoint of the anterior cruciate ligament (ACL); b, midsulcus of tibial spines; c, midpoint of the posterior cruciate ligament (PCL). (C) The slotted line of the tibial osteotomy guide was aligned with the three‐point line, and the distal guide orientated the anterior tibial tendon, which creating a neutral alignment with a right anteroposterior direction.

**Fig. 2:**
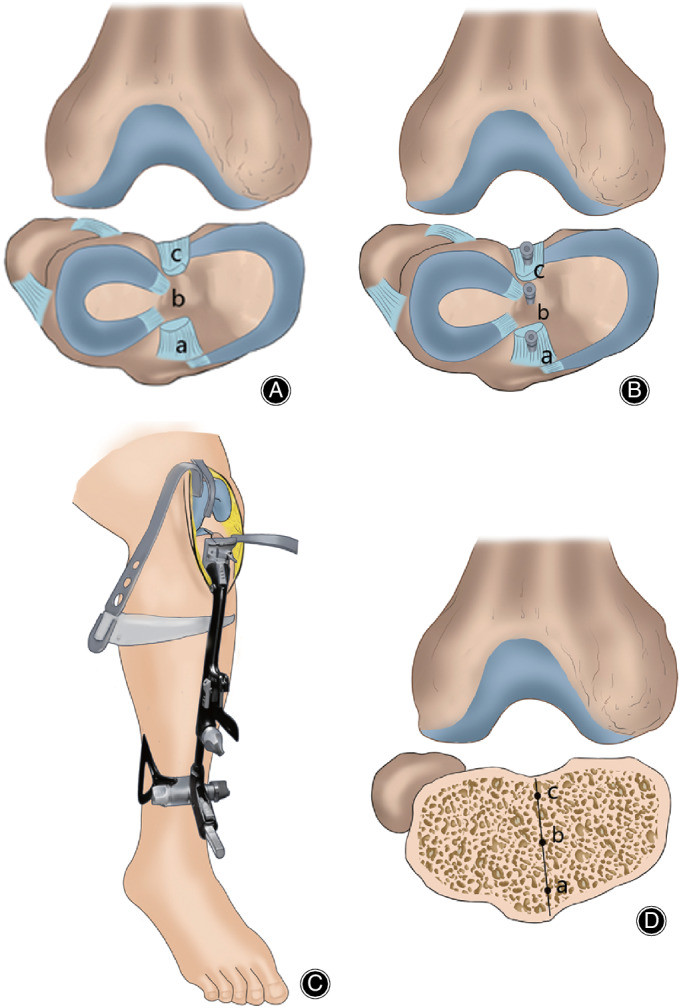
(A) Determination of: a, midpoint of the anterior cruciate ligament (ACL); b, midsulcus of tibial spines; and c, midpoint of the posterior cruciate ligament (PCL). (B) Inserting nails. (C) Alignment of tibial osteotomy guide. (D) Three‐point line on the tibial resection surface.

Tibial resection was performed prior to femoral resection. When the tibial plateau was fully exposed after cutting the ACL and the PCL, we distinguished the midpoint of the PCL, the tibial spines, and the ACL. Then three nails were inserted into the aforementioned reference points to ensure that the landmarks could still be observed after the bone resection (Fig. 1A,B). In most cases (55/64, 86%), these three landmarks could be clearly discerned and we found them almost on the same line (Fig. 1B), which was consistent with a previous 3D CT study^3^. In some cases, the tibial spines (7/64, 11%) or the stumps of the ACL (2/64, 3%) were worn away. We used two landmarks (PCL and tibial spines or PCL and ACL) in such cases. The slotted line of the tibial resection guide was aligned with these points, and the distal tibial guide orientated the anterior tibial tendon, which created a neutral alignment with a right anteroposterior direction (Fig. 1C). A total of 8–10 mm bone was cut, referring to the lateral tibial plateau. The total procedure of the tibial resection is presented in the schematic illustration (Fig. 2).

#### *Femoral Resection and Component Placing*

A femoral intramedullary rod was used for guiding the femoral valgus, cut at 4 to 6°, which was determined by the degrees of difference between the femoral mechanical and anatomical axes. Accessible osteophytes were removed, and the medial soft tissue sleeve was released appropriately to obtain gap balancing in extension. The rotation of the femoral component was determined using a hybrid method that combined measured resection and the gap balancing method^16^. We tried to obtain an optimal rotation that was coincident with the TEA as well as good gap balancing. An appropriately sized tibial component was implanted with cement according to the three‐point line.

#### *Insall's 1/3 Method Group*

In the Insall's 1/3 method group, the coronal resection of the tibia was aligned with the medial 1/3 of the tibial tubercle and the anterior tibial tendon^13^. The rotation of femoral components was also determined using a hybrid method^16^.

### *Outcome Measurement*

The coronal alignment and the axial rotation error were measured on postoperative long‐leg radiographs, and axial CT was undertaken 6 months after the surgery. To determine the intraobserver and the interobserver reliability of the measurements, two of the authors performed blinded measurements. One of the authors repeated measurements with 2 weeks intervals. The average values of the measurements were used for analysis.

#### *Coronal Alignment Error of* *Tibial Component*

**Fig. 3:**
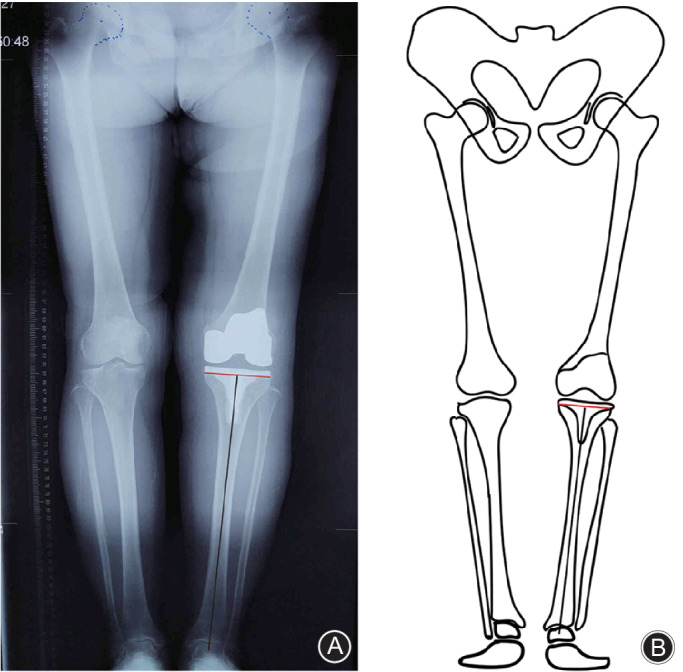
(A) Anteroposterior long‐leg standing radiography shows the tibial coronal alignment. (B) Diagram of radiograph.

The coronal alignment error of the tibial component was determined by the angle between the line parallel to the tibial component platform and the tibial mechanical axis (Fig. 3)^17^, ^18^. Positive values indicated varus resection and negative values valgus resection. Most surgeons believed that to avoid abnormal force on the tibia, the coronal alignment of the tibial component following TKA should be within ±3° of the mechanical axis^9^, ^15^, ^19^. Coronal malalignment of the tibial component can lead to poor functional outcomes and polyethylene wear^9^, ^19^.

#### *Axial Rotation Error of* *Femoral Component*

**Fig. 4:**
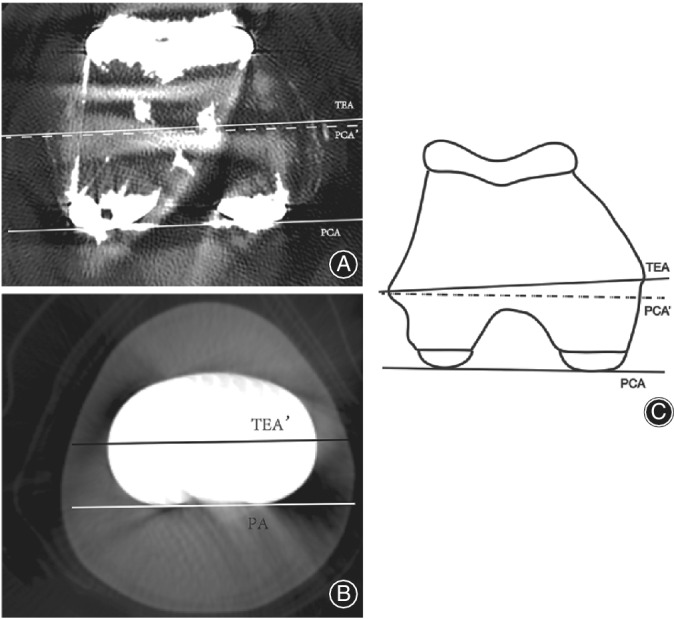
(A) Axial CT image showed rotation error of femoral component. The posterior condylar axis (PCA) was the tangent line of the posterior surface of the femoral component. The transepicondylar axis (TEA) connected the medial and lateral epicondyles. The PCA′ line was parallel to the PCA. The angle between the PCA′ and the TEA represented the axial rotation error of the femoral component. (B) Axial CT image shows the rotation error of the tibial component. The PA line was the tangent line of the posterior surface of tibial component. TEA′ was the projection line of the TEA on tibial scan. The angle between PA and TEA′ represented the axial rotation error of the tibial component.(C) Diagram of femoral axial CT.

The axial rotation error of the femoral component was the intersection angle between the TEA and a line tangent to the posterior edge of the femoral component^1^, ^17^(Fig. 4A,C). Positive values indicated internal rotation relative to TEA. We defined internal/external rotation of components no more than 3° as a safe zone, based on previous studies^10^, ^17^, ^20^. Malrotation of the femoral component in TKA is related to anterior knee pain, abnormal patellar tracking, joint stiffness, and polyethylene wear^20^, ^21^.

#### *Axial Rotation Error of* *Tibial Component*

Similarly, the rotation error of the tibial component was the intersection angle between the TEA and a line tangent to the posterior edge of the tibial component (Fig. 4B)^1^, ^17^. Positive values indicated internal rotation relative to the TEA. We defined internal/external rotation of components of no more than 3° as in a safe zone, based on previous studies^10^, ^17^, ^20^. Malrotation of the tibial component is also related to patellofemoral complications and polyethylene wear^20^, ^21^.

#### *Statistical Analysis*

The reliabilities of intra‐rater and inter‐rater measurements were assessed using intraclass correlation coefficients (ICC), which can be interpreted as: <0.40 poor; 0.40–0.59 fair; 0.60–0.74 good; and 0.75–1.00 excellent^22^. The difference between the rates of gender, side, K‐L grade, and outliers were tested by χ^2^‐test. Quantitative data were compared with independent *t*‐tests. Mann–Whitney *U*‐tests were used when the data did not show a normal distribution. A *P*‐value less than 0.05 was considered significant difference. All statistical analyses were performed with SPSS 21.0 software (IBM, Armonk, NY).

## Results

### *General Information*

A total of 128 knees that underwent TKA from January 2015 to August 2018 were enrolled in this study. In the three‐point method group (64 knees), the Kellgren–Lawrence (K‐L) grade III and IV OA knees were 20 and 44, respectively. In the Insall's traditional method group (64 knees), the K‐L grade III and IV OA knees were 22 and 42, respectively. There were no significant differences in age, gender, side, height, body mass index (BMI), K‐Lgrade, and severity of varus between the two groups (*P* > 0.05) (Table 1). The mean operative time was 73.0 ± 13.5 min in the three‐point method group and 71.9 ± 12.9 min in Insall's traditional method group. The intraoperative blood loss was 50.6 ± 15.6 mL in the three‐point method group and 50.3 ± 15.5 mL in Insall's traditional method group. The mean operative time and intraoperative blood loss did not reach significant difference between the two groups (*P* > 0.05).

### *Reliability*

The intra‐rater reliability was found to be 0.89–0.94 and the inter‐rater reliability was found to be 0.86–0.92. All the measurements showed excellent ICC.

### *Coronal Alignment Error of Tibial Component*

#### *Alignment Error*

**Fig. 5:**
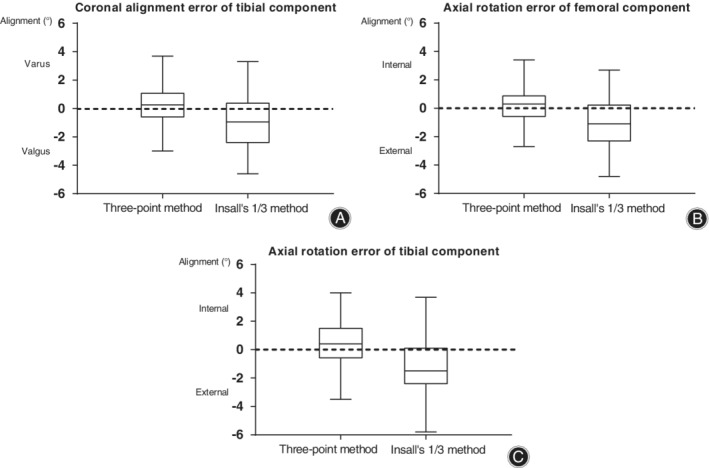
Box plots showing the mean errors of component placing in the two groups. (A) Coronal alignment error of the tibial component. (B) Axial rotation error of the femoral component. (C) Axial rotation error of the tibial component.

The coronal alignment error of the tibial component was 0.2° ± 1.4° in the three‐point method group and −0.9° ± 1.8° in the Insall's traditional method group (Fig. 5A). The mean values reached significant difference between the two groups (*P* < 0.001) (Table 2), and there was no significant difference between grade III and IV OA cases (*P* > 0.05) (Table 3). The mean absolute value in the three‐point method group reduced by 37.3% compared to Insall's traditional method group.

**TABLE 2:** Coronal alignment error and axial rotation error in the two groups (mean ± SD)

	Three‐point method (°)	Insall's method (°)	*P*
Coronal tibial alignment	0.2 ± 1.4	−0.9 ± 1.8	<0.001
Rotational femoral alignment	0.2 ± 1.2	−1.1 ± 1.7	<0.001
Rotational tibial alignment	0.4 ± 1.4	−1.4 ± 1.8	<0.001

**TABLE 3:** Coronal alignment error and axial rotation error in grade III and IV osteoarthritis cases (mean ± SD)

	Three‐point method (°)			Insall's method (°)		
	Grade III (*n* = 20)	Grade IV (*n* = 44)	*P*	Grade III (*n* = 22)	Grade IV (*n* = 42)	*P*
Coronal tibial alignment	0.2 ± 1.5	0.2 ± 1.4	0.94	−0.8 ± 2.0	−1.0 ± 1.7	0.75
Rotational femoral alignment	0.1 ± 1.3	0.3 ± 1.1	0.66	−1.1 ± 1.5	−1.1 ± 1.8	0.96
Rotational tibial alignment	0.3 ± 1.7	0.5 ± 1.3	0.59	−1.3 ± 1.8	−1.4 ± 1.8	0.85

#### *Rate of Outliers*

The coronal malalignment of the tibial component was found in 4 (6%) knees in the three‐point method group and 9 (14%) knees in the Insall's traditional method group (Table 4).

**TABLE 4:** The comparison of the rate of outliers in the two groups

	Three‐point method	Insall's method	*P*
Coronal tibial alignment	6%	14%	0.14
Coronal femoral alignment	3%	14%	0.03
Axial tibial alignment	6%	22%	0.01

### *Axial Rotation Error of Femoral Component*

#### *Axial Rotation Error*

The axial rotation error of the femoral component was 0.2° ± 1.2° in the three‐point method group and − 1.1° ± 1.7° in the Insall's traditional method group (Fig. 5B). The mean values reached significant difference between the two groups (*P* < 0.001) (Table 2), and there was no significant difference between grade III and IV OA cases (*P* > 0.05) (Table 3). The mean absolute value in the three‐point method group reduced by 43.9% compared to Insall's traditional method group.

#### *Rate of Outliers*

The axial malrotation of the femoral component was found in 2 (3%) knees in the three‐point method group and 9 (14%) knees in the Insall's traditional method group. The rate of outliers was significantly lower in the three‐point method group (Table 4).

### *Axial Rotation Error of Tibial Component*

#### *Axial Rotation Error*

The axial rotation of the tibial component was 0.4° ± 1.4° in the three‐point method group and − 1.4° ± 1.8° in the Insall's traditional 1/3 method group (Fig. 5C). The mean values reached significant difference between the two groups (*P* < 0.001) (Table 2), and there was no significant difference between grade III and IV OA cases (*P* > 0.05) (Table 3). The mean absolute value in three‐point method group fell by 35.5% compared to Insall's traditional method group.

#### *Rate of Outliers*

The axial malrotation of the tibial component was found in 4 (6%) knees in the three‐point method group and 14 (22%) knees in the Insall's 1/3 method group. The rate of outliers was significantly lower in the three‐point method group (Table 4).

## Discussion

In the current study, we further discovered that the midpoint of the PCL, the midsulcus line of the tibial spines, and the midpoint of the ACL almost shared one line, and these three anatomical landmarks could complement each other during the operation. We demonstrated that the desired coronal alignment of the tibial component (mean error 0.2° ± 1.4°), the axial rotation of the femoral component (mean error 0.2° ± 1.2°), and the axial rotation of the tibial component (mean error 0.4° ± 1.4°) could be generally established by using this three‐point guiding line. Moreover, we compared the component orientation in patients using Insall's 1/3 method. We found that the rates of rotation outliers were significantly lower in our three‐point method group. The tibial component tended to be rotated externally by using the medial 1/3 of the tibial tubercle as a reference, which was coincident with previous studies^1^, ^3^, ^14^, ^15^.

Identifying the correct anatomical landmarks for tibial resection is very important for the following soft‐tissue releasing and gap balancing procedures^8^, ^16^. Several references have been proposed for the axial and coronal alignment of the tibial component, but a well‐accepted method has not been established^12^, ^23^. The posterior condylar line was reported as an accurate reference for the anteroposterior axis^24^, and the anterior tibial curved cortex of the proximal tibia could also be used as a reference^25^. However, it might be difficult to identify in patients with osteophytes and bony defects of the tibial plateau^26^, ^27^. Akagi *et al*.^1^ describe an anteroposterior axis connecting the medial border of the patellar tendon attachment which was located on the tibial tubercle to the midpoint of the PCL stump. They found that this axis was perpendicular to the TEA in CT scan, and, thus, they recommended this anteroposterior axis as a reference line for the orientation of the tibial component. However, recent studies have shown that the tibial tubercle is not a reliable landmark because of its location variation^28^, ^29^. Dalury^2^ reported using the midsulcus of the tibial spines for orientation of the tibial component. Their methods might also be limited in severe osteoarthritis patients whose tibial spines are worn away.

Previous anatomical study showed that the ACL and the PCL participated in the articulation and rotation of the femorotibial joint, cooperating with the medial and lateral collateral ligaments^30^. Several biomechanical studies have also indicated that the resection of the ACL or the PCL has an influence on tibial rotation^31^, ^32^, ^33^, ^34^. All of these studies imply that the ACL and the PCL contribute to the flexion–extension of the knee and function as stabilizers of the axial rotation of tibia. The APCL line was defined as a line connecting the stumps of midpoints of the ACL and PCL. This direction ensured that the ACL and the PCL had no impingement on the intercondylar notch during knee flexion. It provided a reasonable explanation for APCL formation relative to TEA^3^. In a recent study, Yang *et al*. (2016) showed that the APCL line was almost perpendicular to the TEA.^3^ In addition, they found that the midpoint of the tibial spines nearly coincided with the APCL line, whose perpendicular distance to the APCL line was an average 0.7 mm on the 3D CT model, and this extremely small distance could be almost ignored in clinical practical application^3^. Moreover, in another study, Dalury and Aram^15^ found the line drawn along the midsulcus of the tibial spines and continued in the sagittal plane until the anterior tibia could achieve a neutral resection. Their findings were consistent with the results of the current study^3^, ^15^.

Our study has some limitations. First, the proportion of male patients enrolled in the study is limited, because fewer men than women suffer from OA. Second, the valgus patients are few in number and belong to a rheumatoid arthritis (RA) group. Most of our patients are varus patients with OA. Related research is also being conducted in the RA group, and we are still collecting the data for further analysis. Third, the current study is a consecutive series with a matched‐pair analysis based on age, sex, and BMI between the two groups, and, thus, a more objective assessment is needed. Fourth, the population of our study is limited to Asian subjects and there might be differences between Asian people and those of other races.

### Conclusion

The line connecting the midpoint of the PCL, the midsulcus of tibial spines, and the midpoint of the ACL could be used as the reference for the tibial resection and component placing. By using this method, surgeons could determine the correct tibial resection and component rotation. This method appears to be more accurate than Insall's 1/3 method. For experienced surgeons, who usually prefer no more than one method to guide the tibial resection and component placing, the results of our study may provide a candidate method with little error.
